# Occupational Health Hazards and Control Measures in Government Hospitals: A Cross-Sectional Survey of Nurses' and Nurse Managers' Perspectives

**DOI:** 10.1155/jonm/6657959

**Published:** 2025-03-24

**Authors:** Fathia Ahmed Mersal, Ibrahim Naif Alenezi, Rasmia Abd El Sattar Ali, Faisal Khalaf Alanazi

**Affiliations:** College of Nursing, Northern Border University, Arar, Saudi Arabia

**Keywords:** control measures, nurse managers, nurses, occupational health hazards, work environment

## Abstract

**Background:** Healthcare environments expose workers and patients to hazardous substances, leading to sickness and death. Nurses play a critical role in maintaining patient health and managing occupational risks, including biological, physical, chemical, and psychological hazards, highlighting the need for significant commitment. This study aims to explore the occupational health hazards and control measures among nurses and nurse managers in Saudi Arabia.

**Methods:** A descriptive, cross-sectional study was conducted using a convenience sample of 222 nurses and nurse managers in Northern Border Hospitals. A Google survey questionnaire was utilized for capturing data, including demographic information, occupational health hazards, and control measures.

**Results:** Of the 222 nurses surveyed, 20.3% experienced high ergonomic hazards, 14.4% experienced physical hazards, 14.9% experienced chemical hazards, 17.1% experienced biological hazards, and 34.2% experienced moderate psychological hazards. Regarding safety measures, 86.0% of participants reported high levels of control, 80.6% took proactive safety precautions, and 87.8% adhered to comprehensive control measures. There was little correlation between demographic characteristics, occupational hazards, and control measures. A significant negative association was found between control measures and occupational hazards (*p* value ≤ 0.001).

**Conclusion:** Nurses face a range of occupational hazards, including ergonomic, physical, chemical, biological, and psychological risks. Addressing these hazards is essential for the well-being of nurses, the quality of patient care, and the creation of a healthier work environment. While control measures are generally effective, some healthcare workers lack access to adequate safety protocols. The results indicated a negative correlation between exposure to occupational health hazards and the implementation of control measures.

## 1. Introduction

Occupational hazards are conditions in the work environment that pose potential threats to human health and can be classified as biological or nonbiological [1]. Any work environment has the potential to affect healthcare professionals, either immediately or over time negatively. Given this exposure to various occupational risks, nurses continue to report high rates of injuries and illnesses related to their jobs [2]. The responsibilities and work environment of nurses place them at the forefront of many occupational health hazards, which can lead to acute or long-term health concerns [3].

According to the World Health Organization (WHO), over 59 million people work in healthcare facilities worldwide. About 424,000 people, both Saudis and foreign nationals, work in healthcare settings in the Kingdom of Saudi Arabia (KSA) [4]. Nurses in various healthcare settings face various occupational hazards, including infections from sharp object injuries, exposure to hazardous drugs and radiation, physical injuries, and psychological stress [5].

Nurses and other healthcare professionals are at the forefront of many work-related risks. They are particularly vulnerable to occupational health hazards in the workplace, making nursing a potentially hazardous profession [6]. While some hazards have long existed in the nursing field, they have only recently received appropriate recognition. Other risks have emerged because of rapid advancements within the healthcare field. Nurses frequently provide care in high-risk settings [1].

Nursing requires a significant commitment because it involves the health and lives of individuals. While the profession can be personally fulfilling, it also involves certain health risks [7]. The nature of nursing puts nurses at risk for numerous occupational hazards, including biological, physical, and chemical risks while performing their duties. Their safety depends on their awareness of these risks and knowledge of prevention strategies [8, 9].

Musculoskeletal disorders represent significant occupational health challenges for nurses, as their job requires physical exertion and repeated motions [10]. Studies from various countries have reported an annual incidence rate of 40%–85% for work-related musculoskeletal disorders among nurses [11–13]. These disorders significantly impact the quality of life, leading to productivity loss, absenteeism, decreased job engagement, and lower-quality work output, all of which impact the worker, the company, and society financially [14].

Work-related skin diseases, second only to musculoskeletal disorders in prevalence, primarily take the form of contact dermatitis (85% or more). This allergic and irritant condition, commonly referred to as hand dermatitis, involves the development of lesions because of exposure to irritants and allergens in the workplace [15]. Biological hazards are a significant threat to nurses, exposing them to diseases, such as bacterial infections, tetanus, *tuberculosis,* gonorrhea, and viruses, such as hepatitis, HIV, and COVID-19 [16].

In the delivery of healthcare services, hazardous materials, including environmentally harmful, oxidizing, flammable, irritating, poisonous, carcinogenic, corrosive, infectious, teratogenic, and mutagenic substances, are often used [17]. Nurses may be exposed to these chemicals, particularly during public health crises, risking contact with lethal substances. Ensuring the safety of nurses and other healthcare workers (HCWs) from contamination or exposure to dangerous biological and chemical agents is crucial [18].

Nursing is a high-stress profession, with factors like workload, managerial leadership, staffing, and support resources affecting both the physical and emotional well-being of nurses. Addressing these issues can improve their overall health [19]. Hospital nurses often experience sleep disturbances, fatigue, burnout, and psychological distress because of long hours and heavy workloads. They are also at risk of developing posttraumatic stress symptoms from repeated exposure to traumatic situations [20].

Occupational health nurses play a key role in promoting, improving, and maintaining worker health through prevention, protection, and intervention measures. Their goal is to foster a productive and healthy work environment. The Occupational Health and Safety Act [21] mandates the implementation of medical monitoring and risk management protocols based on data on occupational hazards and safety in the healthcare sector. The Occupational Health and Safety Act lays down the basic regulatory framework for workers' health and safety protection in most industries, including healthcare. However, it requires some specificity in its application to healthcare settings becaaue of the peculiar nature of nursing work environments. Healthcare institutions should implement targeted safety protocols that address the distinctive risks faced by nursing professionals, including exposure to infectious diseases, ergonomic hazards, and psychological stressors [22].

Preventative measures are safety actions taken to reduce or eliminate accidents, illnesses, and injuries among workers to protect them from working dangers. These measures include steps to minimize the likelihood of encountering hazards or efforts to eradicate hazards from the work environment. Given the increased rates of illness and death among workers exposed to occupational hazards, workplaces must adopt preventive measures to safeguard employees [22]. The effectiveness of occupational health and safety (OHS) protocols in preventing and managing infections, particularly in hospitals, is well recognized. These procedures protect HCWs and improve workplace environmental conditions [23].

Research conducted at many government hospitals in KSA found that the yearly incidence of needlestick injuries (NSI) was 3.2 per 100 occupied beds, with nurses being the most affected occupational group [24]. A recent study in a hospital in the Medina region estimated the annual prevalence of NSI among healthcare professionals to be 32% [25].

Despite the extensive literature on occupational health hazards in healthcare settings worldwide, significant gaps remain in understanding these issues within Saudi Arabia's government hospitals, particularly in the Northern Border Region [4]. The Saudi healthcare system faces multifaceted challenges that directly impact the occupational conditions of HCWs, particularly in government hospitals. While 75% of the population is not receiving regular checkups and 60% are overweight or obese [26], HCWs are experiencing increasing workload demands and associated occupational risks. This demographic reality is especially significant in the Northern Border Region, where the burden of chronic conditions creates additional occupational challenges for the healthcare staff. The projected rise in chronic conditions by 2030 suggests increasing workplace demands and potential occupational hazards for HCWs [27–29]. Previous research in the Northern Border Region has indicated general occupational health hazards among HCWs, although significant gaps persist in the recognition of these problems. Significant research in the Northern Border Region revealed essential risk factors and recommended broad strategies [4]; the unique perspectives of nurses and nurse managers in this context remain unexplored.

The literature review for the current study identified a limited number of research studies from KSA focusing on the occupational hazards faced by nurses and nurse managers. These studies addressed both biological and nonbiological hazards, along with their prevalence and associated risk factors. This study aims to explore the experiences of nurses and nurse managers in government hospitals regarding occupational health hazards and control measures. In addition, it seeks to examine the relationship between control measures and occupational hazards.

## 2. Methods

### 2.1. Research Design and Setting

A descriptive, cross-sectional study was performed to explore occupational health hazards and control measures among nurses and nurse managers at Northern Border Hospitals in Saudi Arabia. This design was especially applicable, as it provided the subsequent evaluation of multiple factors at a specific moment, offering a thorough overview of current occupational health problems and existing control measures. Whereas the longitudinal design may yield information about temporal changes, and the mixture of methods could have given greater depth qualitatively, a cross-sectional approach was chosen since it most effectively fulfills the objectives of identifying present methods while simultaneously clarifying broader patterns across many healthcare settings. In addition, this design met both resource and time limitations within the constraint to disrupt clinical health services as little as possible.

### 2.2. Participants

Male and female nursing staff and managers from four major hospitals in the Northern Border Region of Saudi Arabia were recruited for this study, with work experience of 1 year or more. A convenience sampling strategy was utilized to address the issue of low response rates in this study. The study sample consisted of 222 participants, which was determined based on a total population of 1200 nursing personnel. The equation used for calculating the sample size was based on the intended power of 0.80, a type I error (*α*) of 0.05, a type II error (*β*) of 0.2, and a confidence level of 90%.

(1) $$\begin{array}{c} n=\frac{N\times p\;\left(1-p\right)}{\left[\left[N-1\times \left(d^{2\text{⁣}}÷z^{2\text{⁣}}\right)\right]+p\;\left(1-p\right)\right]},\\ 220=\frac{\left(1200\times 0.50\left(1-0.50\right)\right)}{\left[\left[1200-1\times \left(0.05x^{2}÷1.64x^{2}\right)\right]+0.50\left(1-0.50\right)\right]}. \end{array}$$

Convenience sampling was employed to recruit participants from government hospitals. It was practical to recruit the target population within the constraints of hospitals' tight schedules and reach nurses across different shifts and departments. Although probability sampling might yield better representativeness, convenience sampling was the most suitable approach given the timeframe and resources for this study. However, this sampling strategy's limitations regarding possible selection bias and generalization to the larger nursing population at government hospitals are recognized.

### 2.3. Instruments

The data collection for this study consisted of three parts. The first part involved the collection of demographic data, such as age, gender, marital status, hospital name, department working name, current job, years of working experience, educational level, and whether the participant had previously attended a training program.

The second part of the study examined occupational health hazards among nurses and nurse managers by adopting the framework and guidelines outlined by Shamkh, Mohammed, and Al-Abedi [1]. This framework consists of five categories: ergonomics hazards, including entities such as slipping or falling during working, developing varicose veins because of prolonged standing, back pain, joint pain, and allergic reactions from wearing gloves; physical hazards, consisting of items including radiation exposure, x-rays, noise or high voices affecting health during work, and electric shock caused by unqualified tools; chemical hazards, such as poisoning from dealing with chemical solutions and sterilizers, skin disorders caused by the use of chemical solutions and sterilizers, eye, nose, and pharyngeal irritation caused by exposure to cleaning solutions and disinfectants, and burns caused by contact with sterilization equipment and acids; biological hazards, including sharp object-related injuries (e.g., needle sticks), cuts and wounds, direct contact with contaminated specimens/biohazardous materials, airborne diseases, infectious diseases, and other related risks; and psychological hazards, covering items related to stress and work pressures, personal problems with health staff members, and issues with patients or their relatives.

The third part of this study involved using an assessment instrument to examine control measures for reducing OHS hazards among staff nurses and nurse managers. This instrument, adopted from Ndejjo et al. [30], consists of three sections: employer-provided control measures, individual protective measures, and hand hygiene practices. The employer-provided control measures section assesses the availability and effectiveness of organizational-level interventions to mitigate workplace hazards. The individual protective measures section evaluates nurses' and nurse managers' protective strategies and behaviors, including the use of personal protective equipment (PPE) and other safety practices. The hand hygiene practices section focuses on hand hygiene practices in healthcare settings.

The reliability of the questionnaires was assessed by calculating the internal consistency of the items. Cronbach's alpha for occupational hazards was 0.905, while it was 0.853 for control measures. A dichotomous scoring system (yes/no) was used to assess the responses, with each correct response receiving a score of 1 and an incorrect response a score of 0. The total score was calculated to determine the respondents' overall hazard exposure. Implementing a standardized scoring system enhanced the internal consistency and reliability of the questionnaire. Respondents' overall hazard awareness was assessed and classified based on Bloom's cut-off points, which are commonly used in various studies to evaluate knowledge, attitudes, and practices (KAP) regarding health-related issues. Scores ranging from 80% to 100% were categorized as high exposure, scores from 60% to 79% were classified as moderate exposure, and scores below 60% were classified as low exposure [31–33].

### 2.4. Data Collection

Data collection was carried out using a standardized structured questionnaire derived from the work of Shamkh, Mohammed, and Al-Abedi [1] and Ndejjo et al. [30]. A high-reliability score indicates strong internal consistency and stability of the instrument, supporting its appropriateness in achieving the study's aims. Data were collected from December 2023 to March 2024 via an electronic questionnaire distributed through Google Forms. Participants were provided with a link to the complete questionnaire and were informed about the study's purpose and ethical considerations. They were also instructed not to provide personal information until the questionnaire had received joint approval.

### 2.5. Ethical Considerations

Ethical permission for the study was granted from the Local Committee of Bioethics (HAP-09-A-043) at the Northern Border University (approval # 111/23/H) on May 12, 2023. Hospital administrators reached a consensus on the administrative arrangement. Informed permission was electronically acquired from all participants using a secure online platform. The informed consent outlined the study's goal, ensured voluntary participation, affirmed the right to withdraw, and addressed data management. In addition, participants were mandated to explicitly indicate approval by selecting a checkbox to proceed with the survey. Confidentiality was ensured through multiple measures: (a) all data were collected anonymously, without personal identifiers; (b) responses were stored on encrypted servers with limited access; (c) data were aggregated for analysis and reporting, preventing identification of individual responses; and (d) hospital-specific information was coded to preserve institutional anonymity.

### 2.6. Statistical Analysis

The data were analyzed using the Statistical Package for Social Science (SPSS) version 26. Descriptive statistics, including frequencies, percentages, means, and standard deviations, were used for quantitative variables. Statistical significance was tested using appropriate methods. The normality of the data was assessed with the Shapiro–Wilk and Kolmogorov–Smirnov tests, both of which showed that the data did not follow a normal distribution. Consequently, nonparametric tests were applied, including the Mann–Whitney *Z*-test, Kruskal–Wallis H-test, and Spearman correlation test, to analyze the variations in occupational hazard scores based on the sociodemographic features of the participants. A *p* value of less than 0.05 was considered statistically significant.

## 3. Results

### 3.1. Descriptive Results

A total of 222 nurses participated in this study. The majority were aged between 25 and 34 years (45.9%), female (53.2%), and married (78.4%). Most held a bachelor's degree (56.8%) and worked as staff nurses (86.0%). In addition, a significant portion had more than 10 years of experience (44.1%) and had not received training in occupational safety (72.5%). A summary of the participants' demographic characteristics is provided in Table 1.

### 3.2. Exposure to Occupational Health Hazards

Table 2 highlights the percentages of nurses and nurse managers who reported exposure to occupational health hazards across multiple subscales. Among ergonomics hazards, back pain was the most commonly reported issue, with 65.8% of participants indicating exposure. Regarding physical hazards, electric shock caused by unqualified tools was the most prevalent concern, affecting 36.0% of respondents. In the category of chemical hazards, 53.6% of participants reported poisoning because of handling chemical solutions and sterilizers, making it the most frequent chemical-related hazard. For biological hazards, infectious diseases and/or infections were the most reported issues, affecting 42.3% of respondents. Lastly, in the psychological hazards category, the most common issue was psychological reactions to stress and work pressures, which was cited by 35.6% of participants.

Table 3 outlines the control measures implemented to minimize exposure to OHS hazards among nurses and nurse managers. The result shows that 90.5% of nurses and nurse managers received safety education and training on universal precautions, safety tools, equipment, machinery, handwashing, PPE, and proper disposal areas for medical waste. In terms of individual protective measures, 84.2% of respondents received the Bacillus Calmette–Guerin (BCG) vaccine, 89.6% received hepatitis A and B vaccinations, 73.4% reported postexposure prophylaxis, and 91.9% had HIV screening examinations. Regarding handwashing practices, over 90% of participants practiced handwashing after meals, using the toilet, handling soiled materials, and after each procedure.

Table 4 shows that 20.3% of nurses reported high exposure to ergonomic hazards, while physical and chemical hazards were reported by 14.4% and 14.9% of respondents, respectively. Biological hazards affected 17.1% of participants, and 34.2% reported moderate exposure to psychological hazards. Among these, 6.3% experienced significant stressors and challenges impacting their mental well-being. Participants have implemented effective measures, with 86.0% reporting high levels of control. In addition, 80.6% of participants took proactive precautions to protect themselves. Handwashing practices were also high, with 88.3% of participants adhering to recommended practices. Overall, 87.8% of respondents reported high levels of control measures.

The differences in self-reported occupational health hazards and control measures between male and female nurses, and nurses and nurse managers/heads are presented in Supporting file 1. Female participants reported higher exposure rates of ergonomic hazards, at 23.7%, while for males, the rate was 16.3%. Regarding control measures, the implementation rates for both gender groups were high. Handwashing practices were the highest in females, 89.8%, followed by males, 86.5%. In addition, ergonomic risks exhibited comparable high exposure rates among nurses (20.4%) and nurse managers (19.4%). Concerning control measures, both groups indicated strong implementation rates. Staff nurses adhered more to handwashing protocols (89.5%, high implementation) than nurse managers (80.6%) (see Supporting file 1 and Tables 1 and 2).

Table 5 shows a negative correlation between occupational health hazard exposure, ergonomics hazards, physical hazards, chemical hazards, biological hazards, psychological hazards, and control measures among nurses. The result indicates that nurses with higher control measures are less exposed to occupational health hazards (rho = −0.356, *p* < 0.001).

The relationship between demographic characteristics and exposure to occupational health hazards and control measures is presented in Supporting File 1 and Table 3. Concerning the relationship between demographic characteristics and exposure to occupational health hazards, the results show that nurses and nurse managers aged 45 and above had higher occupational hazard scores than younger participants. However, this difference was not statistically significant. Participants with higher educational levels had lower occupational hazard scores, while the highest scores were observed in critical care units. The study found no significant difference in occupational hazards based on years of experience (*p* > 0.05).

In terms of the relationship between demographic characteristics and control measures among nurses, there were no significant differences in control measure scores across different age groups, genders, marital status, educational levels, working departments, job titles, hospital names, or years of experience. However, a significant difference was found in the mean scores of control measures for individuals who had undergone training programs (*p* ≤ 0.05).

## 4. Discussion

This study aimed to explore the experiences of nurses and nurse managers in government hospitals regarding occupational health hazards and control measures, as well as to examine the relationship between the occurrence of occupational hazards and control measures. The results of this study revealed valuable contributions to understanding nurses' occupational hazards and how control measures allay these risks. Identifying the different hazards in this study outlines the multifaceted nature of nurses' workplace hazards. Each of these types of hazards carries different challenges. There are musculoskeletal disorders from ergonomic exposure, physical hazards from radiation exposure, and psychological stress, as it pertains to mental health and employment performance. Healthcare environments are characterized by significant exposure to hazardous substances, which contribute to sickness and death among both patients and healthcare professionals [34]. Nursing is a vital profession that requires substantial dedication, and nurses face numerous occupational hazards while fulfilling their duties [16]. Biological, physical, chemical, and psychological hazards are common in healthcare settings, as emphasized by Walton and Rogers [35], reinforcing the importance of occupational safety for nurses.

The study's findings revealed that a negative correlation between occupational hazard exposure and the effectiveness of control measures (*p* value < 0.001) underscores a critical dynamic in workplace safety, particularly in high-risk environments such as healthcare settings. This relationship highlights the critical role of proactive interventions in modifying risks and fostering safer working conditions for nursing professionals. Effective control mechanisms minimize exposure to physical hazards, including ergonomic strain and chemical agents, as well as psychosocial stressors, such as workplace violence and emotional exhaustion, thereby protecting the physical and mental health of nurses. This combination of safeguards is vital, as mental well-being is increasingly acknowledged as a fundamental aspect of occupational health, directly affecting job performance, retention, and the quality of patient treatment [36]. The findings correspond with existing studies highlighting the reciprocal relationship between occupational safety and healthcare outcomes, indicating that expenditures in workplace safety benefit both employee welfare and the performance of the organization.

An essential inference of this study is the necessity of comprehensive training programs that equip nurses with the knowledge and skills to recognize, assess, and mitigate workplace hazards. Training in safety protocols, hazard identification, and the proper use of PPE is essential for fostering a culture of safety within healthcare institutions. Such programs not only empower nurses to implement control measures effectively but also promote collective responsibility for workplace safety [37]. Moreover, the integration of evidence-based practices and continuous professional development in occupational health can further strengthen the resilience of nursing staff, reducing the incidence of work-related injuries, burnout, and mental health disorders.

The study findings emphasized the mental health and well-being of participants. In contrast, psychological hazards pose the most significant risks to nurses, as they can have long-lasting effects on both nurses and the healthcare system. Addressing these hazards through targeted interventions and support systems is crucial for a healthier work environment and high-quality patient care. Mental health is crucial for nurses' well-being and job performance, as they face psychological hazards in demanding jobs. High stress levels can lead to burnout, anxiety, and depression, negatively impacting patient care and healthcare outcomes. Addressing mental health issues is essential for nurses' overall well-being, as psychologically disturbed nurses are less likely to perform effectively, leading to job dissatisfaction, absenteeism, and turnover. Evidence-based interventions like counseling services and stress management programs can help create a supportive work environment, value nurses, and build resilience [38].

However, the report also reveals a significant difference between perceived effectiveness and actual use and availability of control measures. The findings indicate that although a more significant portion of nurses believe control measures are effective in most instances, protection measure accessibility has not reached or provided protection for many on-the-job workers. This discrepancy underlines the fact that there is a real need for continuous improvement in safety measures implemented in various healthcare settings. According to the negative correlation between exposure to occupational health hazards and efficiency of control measures, this can be achieved by introducing improved measures, which will make it possible to reduce risks to the health of nurses significantly. Overcoming such barriers will require multidimensional intervention, including mental discussion, access to confidential advice, and institutional policies prioritizing psychological security. For instance, care providers can use a participatory model for security planning, with nurses participating in developing and testing controls for usability and effectiveness in real-life settings. In addition, the use of technological innovation, such as electronic platforms for mental care and real-time danger tracking, could make such interventions larger in impact and easier to access [3, 39].

The study also explored the relationship between demographic characteristics and the effectiveness of occupational hazard control measures. The findings revealed no significant differences in occupational hazard scores based on demographic factors, including age, gender, marital status, education level, job title, hospital affiliation, or years of experience. However, individuals who had participated in training programs exhibited slightly higher mean scores for control measures, suggesting that training plays a crucial role in improving awareness and adherence to safety protocols. This aligns with findings by Shamkh, Mohammed, and Al-Abedi [1] and Koskei, Mburu, and Nyamongo [40], who observed that trained HCWs were less likely to experience work-related injuries. The lack of significant demographic differences suggests that occupational hazard and control measures are universally applicable across different groups of nurses and nurse managers. However, it is crucial to ensure that all staff, regardless of their background, have access to continuous training and education to enhance the effectiveness of these control measures and reduce the risks of occupational hazards.

The broader implications of these findings extend beyond individual well-being to encompass organizational and systemic outcomes. By reducing occupational hazard exposure, healthcare institutions can mitigate the financial and operational burdens associated with workplace injuries, such as absenteeism, turnover, and reduced productivity. Furthermore, safer working environments contribute to higher levels of job satisfaction and retention among nursing staff, addressing longstanding challenges in workforce sustainability [41]. These benefits are particularly significant in the context of global nursing shortages, where the retention of skilled professionals is paramount to maintaining healthcare delivery standards.

This study also contributes to the growing body of literature on the intersection of occupational health and patient care quality. The findings reinforce the perception that well-being of nurses is inextricably linked to patient outcomes, as healthier and competent nurses are better equipped to provide compassionate, effective care. This alignment with the Quadruple Aim framework, which emphasizes improving patient experience, population health, cost-effectiveness, and provider well-being, underscores the strategic importance of occupational safety initiatives in healthcare reform [42]. By prioritizing the health and safety of nursing staff, healthcare organizations can create a virtuous cycle that enhances both employee satisfaction and patient care quality.

## 5. Limitations

This study on occupational health hazards among nurses has several limitations. The sample size and geographic scope may not accurately represent the diverse population of nurses in different healthcare settings or regions. In addition, self-reported data from nurses may introduce potential reporting biases, such as recall bias, social desirability bias, or subjective interpretations of questions. The study's cross-sectional design limits our ability to demonstrate causal links between occupational hazards and their effects on nurses' health and well-being. Longitudinal studies would be beneficial in understanding the temporal dynamics and long-term implications of occupational hazards among nurses. Therefore, caution should be exercised when extrapolating the results to other contexts.

## 6. Conclusion

This study emphasized the necessity for ongoing improvement in workplace safety protocols in healthcare environments, with a specific focus on mental health as a part of workplace wellness. The opposite relation between hazard exposure and controls underlines the potential for proactive interventions in minimizing risks and enhancing outcomes for both nurses and patients. Long-term studies in the future must assess the impact of increased protocols for workplace safety in workforce resilience and patient care and the role played by organizational culture and leadership in creating a workplace with a safety orientation. By closing these gaps, healthcare organizations can proceed toward attaining both outcomes of protecting their workforce and providing high-quality patient care, with both physical and mental well-being prioritized in striving for workplace safety.

### 6.1. Implications for Future Research

This study emphasizes the need for continuous nurse education and training, organizational support, and safety measures. It suggests that age and educational levels may influence exposure to occupational hazards. Future research should include longitudinal studies, evaluating interventions, exploring age-related vulnerability, examining the impact of hazards on patient outcomes, investigating organizational factors, identifying emerging hazards, conducting comparative studies, and integrating interventions into nursing education. These approaches would help to deepen the understanding of occupational health hazards, inform evidence-based interventions, and promote safer work environments, ultimately enhancing patient care quality and creating a sustainable healthcare system.

## Figures and Tables

**Table 1:** Summary of participants' demographic characteristics (*N* = 222).

Demographic characteristics	No	%
Age/years		
< 25	37	16.7
25–34	102	45.9
35–44	72	32.4
≥ 45	11	5.0
Gender		
Male	104	46.8
Female	118	53.2
Marital status		
Married	174	78.4
Unmarried	48	21.6
Educational level		
Diploma	69	31.1
Bachelor	126	56.8
Master	25	11.3
PhD	2	0.8
Work department		
Critical care units	83	37.4
Inpatient ward	102	45.9
Administration and quality	37	16.7
Job title		
Staff nurse	191	86.0
Head nurse	23	10.4
Nurse manager	8	3.6
Hospital name		
Al Alamir Abdulaziz bin Musa'id	91	41.0
Maternity and child hospital	39	17.6
Al Burj medical hospital and cardiac hospital	92	41.4
Training program about occupational safety		
No	161	72.5
Yes	61	27.5
Years of experience		
< 5 years	83	37.4
5–10 years	41	18.5
> 10 years	98	44.1

**Table 2:** Occupational health hazards among nurses and nurse managers (*N* = 222).

Items	Yes	
	*N*	%
Ergonomics hazards		
Slipping or falling during working	77	34.7
Varicose veins in legs because of prolonged standing	61	27.5
Back pain	146	65.8
Joint pain	55	24.8
Hand allergies as a result of wearing gloves	80	36.0
Physical hazards		
Radiation, for example, x-ray	77	34.7
Noises or high voices affecting health during work	68	30.6
Electric shock caused by unqualified tools	80	36.0
Chemical hazards		
Poisoning because of dealing with chemical solutions and sterilizers	119	53.6
Skin diseases because of the use of chemical solutions and sterilizers	90	40.5
Irritation from cleaning agents and disinfectants that affects the eyes, nose, and throat	80	36.0
Burns from contact with sterilization equipment and acids during work	67	30.2
Biological hazards		
Sharp-related injuries (e.g., needle sticks)	78	35.1
Cuts and wounds	78	35.1
Direct contact with biohazardous items or contaminated specimens	64	28.8
Airborne diseases	57	25.7
Infectious diseases and/or infections	94	42.3
Others (vector-borne diseases, blood-borne infections, and bioterrorism)	43	19.4
Psychological hazards		
Psychological reactions because of stress and work pressure	79	35.6
Personal problems with the health staff members	28	12.6
Personal problems with the patients or their relatives	11	5.0

**Table 3:** Control measures for occupational health and safety hazards.

Items	Yes	
	*N*	%
Control measures provided by employers		
Safety education and training on all universal precautions	201	90.5
Safety tools, equipment, and machinery	189	85.1
Training in all machinery and equipment used	204	91.9
Training on how to wash hands	194	87.4
Personal set of personal protective equipment	209	94.1
Separate areas and containers to dispose of medical waste	198	89.2
Individual protective measures		
BCG vaccination	187	84.2
Hepatitis A vaccination	199	89.6
Hepatitis B vaccination	187	84.2
Provision of postexposure prophylaxis	163	73.4
Received HIV screening examination	204	91.9
Handwashing practices		
After handling soiled materials	205	92.3
When hands are dirty	212	95.5
Before and after meals	209	94.1
After using the toilet	210	94.6
After removing gloves	192	86.5
Before and after every procedure	211	95.0
Before and after handling each patient	196	88.3
After handling biological samples	211	95.0

**Table 4:** Total occupational health hazards and control measures among the participants.

Items	High exposure		Moderate exposure		Low exposure	
	*N*	%	*N*	%	*N*	%
Occupational health hazards						
Ergonomics hazards	45	20.3	25	11.3	152	68.5
Physical hazards	32	14.4	35	15.8	155	69.8
Chemical hazards	33	14.9	38	17.1	151	68.0
Biological hazards	38	17.1	17	7.7	167	75.2
Psychological hazards	14	6.3	76	34.2	132	59.5
Control measures						
Control measures provided by employers	191	86.0	13	5.9	18	8.1
Individual protective measures	179	80.6	22	9.9	21	9.5
Handwashing practices	196	88.3	17	7.7	9	4.1

**Table 5:** Correlation between occupational health hazards and control measures among nurses (*N* = 222).

Items	Control measures	
	Spearman's rho	*p* value
Total exposure to occupational health hazards	−0.356⁣^∗∗^	< 0.001
Total ergonomics hazards	−0.207⁣^∗∗^	0.002
Total physical hazards	−0.271⁣^∗∗^	< 0.001
Total chemical hazards	−0.270⁣^∗∗^	< 0.001
Total biological hazards	−0.301⁣^∗∗^	< 0.001
Total psychological hazards	−0.296⁣^∗∗^	< 0.001

^∗∗^Correlation is significant at the 0.01 level (two-tailed).

## Data Availability

The data that support the findings of this study are available on request from the corresponding author. The data are not publicly available due to privacy or ethical restrictions.

## Supporting Information

Supporting file 1 includes tables on total self-reported exposure to occupational health hazards and control measures, categorized by gender (Table 1) and job title (Table 2), as well as the relationship between demographic characteristics, exposure to occupational health hazards, and control measures among nurses (Table 3).
