# Supplementary material for: Occupational Health Hazards and Control Measures in Government Hospitals: A Cross-Sectional Survey of Nurses' and Nurse Managers' Perspectives
Source: J Nurs Manag. 2025 Mar 24;2025:6657959. doi: 10.1155/jonm/6657959 (PMC11957864; doi:10.1155/jonm/6657959)
Supplement: Supporting Information — Additional supporting information can be found online in the Supporting Information section. [file 6657959.f1.docx]

**Supplementary File 1:**

**Table 1: Total Self-Reported Exposure and Control Measures to Different Occupational Health Hazards by Gender**

| **Items** | **Male** | | | | | | **Female** | | | | | |
| --- | --- | --- | --- | --- | --- | --- | --- | --- | --- | --- | --- | --- |
|  | **High**  **Exposure** | | **Moderate**  **Exposure** | | **Low**  **Exposure** | | **High**  **Exposure** | | **Moderate**  **Exposure** | | **Low**  **Exposure** | |
|  | **N** | **%** | **N** | **%** | **N** | **%** | **N** | **%** | **N** | **%** | **N** | **%** |
| **Occupational Health Hazards** |  |  |  |  |  |  |  |  |  |  |  |  |
| Ergonomics hazards | 17 | 16.3 | 14 | 13.5 | 73 | 70.2 | 28 | 23.7 | 11 | 9.3 | 79 | 66.9 |
| Physical hazards | 3 | 2.9 | 9 | 8.7 | 92 | 88.5 | 1 | .8 | 7 | 5.9 | 110 | 93.2 |
| Chemical hazards | 16 | 15.4 | 16 | 15.4 | 72 | 69.2 | 17 | 14.4 | 22 | 18.6 | 79 | 66.9 |
| Biological hazards | 19 | 18.3 | 5 | 4.8 | 80 | 76.9 | 19 | 16.1 | 12 | 10.2 | 87 | 73.7 |
| Psychological hazards | 3 | 2.9 | 9 | 8.7 | 92 | 88.5 | 1 | .8 | 7 | 5.9 | 110 | 93.2 |
| Total hazards | 8 | 7.7 | 15 | 14.4 | 81 | 77.9 | 8 | 6.8 | 19 | 16.1 | 91 | 77.1 |
| **Control measures** |  |  |  |  |  |  |  |  |  |  |  |  |
| Control measures provided by employers | 91 | 87.5 | 5 | 4.8 | 8 | 7.7 | 100 | 84.7 | 8 | 6.8 | 10 | 8.5 |
| Individual protective measures | 88 | 84.6 | 9 | 8.7 | 7 | 6.7 | 91 | 77.1 | 13 | 11.0 | 14 | 11.9 |
| Handwashing practices | 90 | 86.5 | 8 | 7.7 | 6 | 5.8 | 106 | 89.8 | 9 | 7.6 | 3 | 2.5 |
| Total control measures | 94 | 90.4 | 5 | 4.8 | 5 | 4.8 | 101 | 85.6 | 13 | 11.0 | 4 | 3.4 |

Table 1 indicates differences in self-reported occupational health hazards and control measures between male and female healthcare workers. Female participants reported higher exposure rates of ergonomic hazards, at 23.7%, while for males, the rate was 16.3%.

Biological hazards were the most prevalent high-exposure risk in both genders, with male participants slightly higher at 18.3%, compared to females at 16.1%. On the other hand, physical and psychological hazards have the lowest high-exposure rates for both genders; the percentage for both types of hazards was particularly low in female participants at 0.8%, compared to males at 2.9%.

Regarding control measures, the implementation rates for both gender groups were high. Handwashing practices were the highest in females, 89.8%, followed by males, 86.5%. Employer-provided control measures were highly reported at 87.5% males and 84.7% females. Individual protective measures were relatively lower but still quite high, with 84.6% for males and 77.1% for females. The cumulative analysis of total hazards revealed similar patterns between genders, with about three-quarters of male and female participants reporting low exposure levels of 77.9% and 77.1%, respectively. Correspondingly, total control measures showed high implementation rates across both groups, with males reporting slightly higher rates of 90.4% compared to females at 85.6%.

**Table 2: Total Self-Reported Exposure and Control Measures to Different Occupational Health Hazards**

| **Items** | **Nurses** | | | | | | **Head Nurses and managers** | | | | | |
| --- | --- | --- | --- | --- | --- | --- | --- | --- | --- | --- | --- | --- |
|  | **High**  **Exposure** | | **Moderate**  **Exposure** | | **Low**  **Exposure** | | **High**  **Exposure** | | **Moderate**  **Exposure** | | **Low**  **Exposure** | |
|  | **N** | **%** | **N** | **%** | **N** | **%** | **N** | **%** | **N** | **%** | **N** | **%** |
| **Occupational Health Hazards** |  |  |  |  |  |  |  |  |  |  |  |  |
| Ergonomics hazards | 39 | 20.4 | 19 | 9.9 | 133 | 69.6 | 6 | 19.4 | 6 | 19.4 | 19 | 61.3 |
| Physical hazards | 3 | 1.6 | 12 | 6.3 | 176 | 92.1 | 1 | 3.2 | 4 | 12.9 | 26 | 83.9 |
| Chemical hazards | 25 | 13.1 | 34 | 17.8 | 132 | 69.1 | 8 | 25.8 | 4 | 12.9 | 19 | 61.3 |
| Biological hazards | 32 | 16.8 | 15 | 7.9 | 144 | 75.4 | 6 | 19.4 | 2 | 6.5 | 23 | 74.2 |
| Psychological hazards | 3 | 1.6 | 12 | 6.3 | 176 | 92.1 | 1 | 3.2 | 4 | 12.9 | 26 | 83.9 |
| Total hazards | 13 | 6.8 | 29 | 15.2 | 149 | 78.0 | 3 | 9.7 | 5 | 16.1 | 23 | 74.2 |
| **Control measures** |  |  |  |  |  |  |  |  |  |  |  |  |
| Control measures provided by employers | 165 | 86.4 | 11 | 5.8 | 15 | 7.9 | 26 | 83.9 | 2 | 6.5 | 3 | 9.7 |
| Individual protective measures | 153 | 80.1 | 21 | 11.0 | 17 | 8.9 | 26 | 83.9 | 1 | 3.2 | 4 | 12.9 |
| Handwashing practices | 171 | 89.5 | 14 | 7.3 | 6 | 3.1 | 25 | 80.6 | 3 | 9.7 | 3 | 9.7 |
| Total control measures | 169 | 88.5 | 16 | 8.4 | 6 | 3.1 | 26 | 83.9 | 2 | 6.5 | 3 | 9.7 |

Table 2 describes the occupational health risks that indicate considerable disparities between nurses and nurse managers/head nurses. Ergonomic risks exhibited comparable high exposure rates among nurses (20.4%) and nurse managers (19.4%). The most substantial differential across positions was noted in chemical risks, with a markedly higher incidence of high exposures among nurse managers compared to staff nurses: 25.8% versus 13.1%. Nurse managers reported a greater incidence of biological hazards than nurses, with 19.4% compared to 16.8% for high exposure. Both physical and psychological dangers led to the lowest high-exposure rates for the two occupational categories, exhibiting nearly comparable patterns: nurses at 1.6% and managers at 3.2%.

Concerning control measures, both groups indicated strong implementation rates. Staff nurses adhered more to handwashing protocols (89.5% high implementation) than nurse managers (80.6%). Control measures given by employers were similarly employed in both categories (nurses: 86.4%, managers: 83.9%). The application rates of individual preventive measures were similarly high among nurses (80.1%) and managers (83.9%). The overall hazards revealed a similar pattern demonstrating primarily modest exposure levels (nurses: 78.0%, managers: 74.2%). The overall control measures showed a considerable degree of implementation for both groups, with nurses reporting higher rates (88.5%) than managers (83.9%).

**Table 3: Relationship between Demographic Characteristics, Exposure to Occupational Health Hazards, and** **Control Measures among Nurses (N= 222)**

| **Demographic Characteristics** | **No** | **%** | **Occupational**  **hazards** | | **Z/H-test**  **(P-value)** | **Control measures** | | **Z/H-test**  **(P-value)** |
| --- | --- | --- | --- | --- | --- | --- | --- | --- |
|  |  |  | **Mean** | **SD** |  | **Mean** | **SD** |  |
| ***Age/ years*** |  |  |  |  | 2.741 (.433) |  |  | 4.228 (.238) |
| <25 | 37 | 16.7 | 7.81 | 6.23 |  | 18.41 | 2.24 |  |
| 25–34 | 102 | 45.9 | 6.72 | 5.39 |  | 18.08 | 2.65 |  |
| 35–44 | 72 | 32.4 | 7.82 | 6.19 |  | 17.88 | 3.89 |  |
| ≥45 | 11 | 5.0 | 10.09 | 7.25 |  | 15.82 | 5.78 |  |
| ***Gender*** |  |  |  |  | –1.083 (.279) |  |  | –.524 (.600) |
| Male | 104 | 46.8 | 7.03 | 6.04 |  | 18.04 | 3.15 |  |
| Female | 118 | 53.2 | 7.77 | 5.80 |  | 17.88 | 3.37 |  |
| ***Marital status*** |  |  |  |  | –1.345 (.179) |  |  | –1.923 (.054) |
| Married | 174 | 78.4 | 7.10 | 5.71 |  | 18.29 | 2.36 |  |
| Unmarried | 48 | 21.6 | 8.60 | 6.52 |  | 16.73 | 5.25 |  |
| ***Educational level*** |  |  |  |  | 1.222 (.748) |  |  | 2.390 (.496) |
| Diploma | 69 | 31.1 | 7.99 | 6.35 |  | 18.32 | 2.66 |  |
| Bachelor | 126 | 56.8 | 7.11 | 5.54 |  | 18.20 | 2.27 |  |
| Master | 25 | 11.3 | 7.76 | 6.70 |  | 15.68 | 6.71 |  |
| PhD | 2 | 0.8 | 3.50 | 3.54 |  | 18.50 | 0.71 |  |
| ***Working department*** |  |  |  |  | 3.536 (.171) |  |  | 5.363 (.068) |
| Critical care units | 83 | 37.4 | 8.27 | 5.82 |  | 18.13 | 2.37 |  |
| Inpatient ward | 102 | 45.9 | 6.89 | 5.93 |  | 17.52 | 3.96 |  |
| Administration and quality | 37 | 16.7 | 7.00 | 6.01 |  | 18.76 | 2.72 |  |
| ***Job title*** |  |  |  |  | 1.059 (.589) |  |  | 1.477 (.478) |
| Staff Nurse | 191 | 86.0 | 7.28 | 5.90 |  | 18.08 | 2.99 |  |
| Head nurse | 23 | 10.4 | 8.30 | 6.02 |  | 17.22 | 4.73 |  |
| Nurse manager | 8 | 3.6 | 8.38 | 6.30 |  | 17.13 | 4.55 |  |
| ***Hospital name*** |  |  |  |  | .308 (.857) |  |  | 2.150 (.341) |
| AlAmir Abdulaziz bin Musaid | 91 | 41.0 | 7.41 | 6.26 |  | 17.70 | 3.37 |  |
| Maternity and Child Hospital | 39 | 17.6 | 7.77 | 5.75 |  | 18.00 | 4.35 |  |
| Al Burj Medical Hospital and Cardiac Hospital | 92 | 41.4 | 7.29 | 5.68 |  | 18.18 | 2.57 |  |
| ***Training program about occupational safety*** |  |  |  |  | –.651 (.515) |  |  | –2.215 (.027) * |
| No | 161 | 72.5 | 6.90 | 5.61 |  | 16.90 | 4.76 |  |
| Yes | 61 | 27.5 | 7.62 | 6.03 |  | 18.35 | 2.38 |  |
| ***Years of experience*** |  |  |  |  | 1.671 (.434) |  |  | .866 (.648) |
| <5 years | 83 | 37.4 | 7.70 | 5.78 |  | 18.13 | 2.46 |  |
| 5–10 years | 41 | 18.5 | 6.29 | 5.33 |  | 17.39 | 4.14 |  |
| >10 years | 98 | 44.1 | 7.66 | 6.25 |  | 18.04 | 3.45 |  |
